# Yoda1 opens the lymphatic path for craniosynostosis therapy

**DOI:** 10.1172/JCI176858

**Published:** 2024-02-15

**Authors:** Aleksanteri Aspelund, Kari Alitalo

**Affiliations:** 1 Wihuri Research Institute, Biomedicum Helsinki, Helsinki, Finland.; 2 Department of Ophthalmology, Helsinki University Hospital, Helsinki, Finland.; 3 Translational Cancer Medicine Program, University of Helsinki, Helsinki, Finland.

## Abstract

The rediscovery of meningeal lymphatic vessels (MLVs) has sparked research interest in their function in numerous neurological pathologies. Craniosynostosis (CS) is caused by a premature fusion of cranial sutures during development. In this issue of the *JCI*, Matrongolo and colleagues show that *Twist1*-haploinsufficient mice that develop CS exhibit raised intracranial pressure, diminished cerebrospinal fluid (CSF) outflow, and impaired paravascular CSF-brain flow; all features that were associated with MLV defects and exacerbated pathology in mouse models of Alzheimer’s disease. Activation of the mechanosensor Piezo1 with Yoda1 restored MLV function and CSF perfusion in CS models and in aged mice, opening an avenue for further development of therapeutics.

## Discovery of meningeal lymphatic vessels

The recent rediscovery of meningeal lymphatic vessels (MLVs) as a CSF drainage route has generated substantial interest in understanding their role in the maintenance of homeostasis in the CNS in health and disease (1, 2). Prior to these findings, CSF was thought to drain out of the CNS mainly through dural cerebral veins (CVs) and perineural spaces that lead to extracranial lymphatic vessels (LVs). While it still holds true that the brain parenchyma lacks LVs, the identification of MLVs and their contribution to the clearance of CSF and to trafficking of immune cells from the CNS has challenged the traditional perspective. Ongoing research is actively exploring the role of MLV drainage, pathological alterations in MLVs, and the therapeutic manipulation of MLVs in aging and mouse models of various CNS pathologies including brain tumors, Alzheimer’s disease (AD), neuroinflammation, stroke, traumatic brain injury (TBI), CNS infections, and cerebral edema (3).

## Craniosynostosis pathology

During the early stages of human infancy or postnatal stages in mice, the skull vault undergoes rapid expansion to accommodate the growing brain. This process is associated with an increase in blood plasma and CSF volumes as well as intracranial pressure (ICP). The primary expansion of the skull takes place at the cranial sutures situated between the bones, which coincides also with the development of the major MLVs (4–6).

Craniosynostosis (CS) is characterized by premature fusion of one or more cranial sutures, leading to a distortion in the developmental shape of the skull. Depending on the severity, CS may be complicated by increased ICP, airway obstruction, feeding difficulties, and exorbitism (6). Over 20% of CS cases result from single gene mutations or chromosomal abnormalities. *TWIST1* haploinsufficiency is a common defect in CS associated with Saethre-Chotzen syndrome. *TWIST1* encodes a basic helix-loop-helix transcription factor, which, among many other functions, regulates the expression of fibroblast growth factor receptors (FGFRs). Gain-of-function mutations of FGFR2 and FGFR3 are also commonly found in Alpers and Muenke-type CS syndromes, respectively (6).

Increased ICP is a major concern in CS. Normal ICP in humans is around 10 mmHg, but it may increase because of TBI, stroke, brain tumors, or other pathologies. When ICP increases above 20 mmHg, it can impair CNS perfusion, resulting in neuronal damage (7). ICP increase in CS has been proposed to arise via hydrocephalus, cranio-cerebral disproportion, airway obstruction, and venous hypertension, but their relative contributions are unclear (8). Recently, CS and mutations in *TWIST1* were also shown to cause CV malformations, which may result in increased ICP. Such CV malformations were also observed in a more profound mouse model of Saethre-Chotzen syndrome, in which one *Twist1* allele possesses a loss-of-function mutation and the other *Twist1* allele is deleted in the periosteal dura and sutures (known as *Twist1^fl/–^;Sm22a-Cre* mice) (9). Mechanistically, it appears that the CV malformations develop as a secondary consequence of the skull malformations via a crosstalk among the skull progenitor cells (SPCs), dura, and CV endothelial cells, involving BMP signaling (9). The extent to which MLVs regulate ICP is not yet known, but the Tischfield group has previously shown that *Twist1*-related CS models also exhibit regionally impaired MLV growth and expansion and impaired CSF drainage to deep cervical lymph nodes (dcLNs) (10), suggesting that defective MLVs may contribute to increased ICP. However, despite a lack of MLVs in superior and basal parts of the skull, ICP is normal in the *K14-VEGFR3-Ig* transgenic mice in which the lymphangiogenic VEGF-C/D growth factor signaling pathway is inhibited (2). On the other hand, increased ICP was shown to contribute to MLV dysfunction in TBI (11). Thus, it was not known to what extent decreased skull size, impaired CV outflow, and defective MLVs functionally interact in the context of increased ICP.

## Meningeal lymphatic defects in *Twist1*-mutant mice

**Figure 1:**
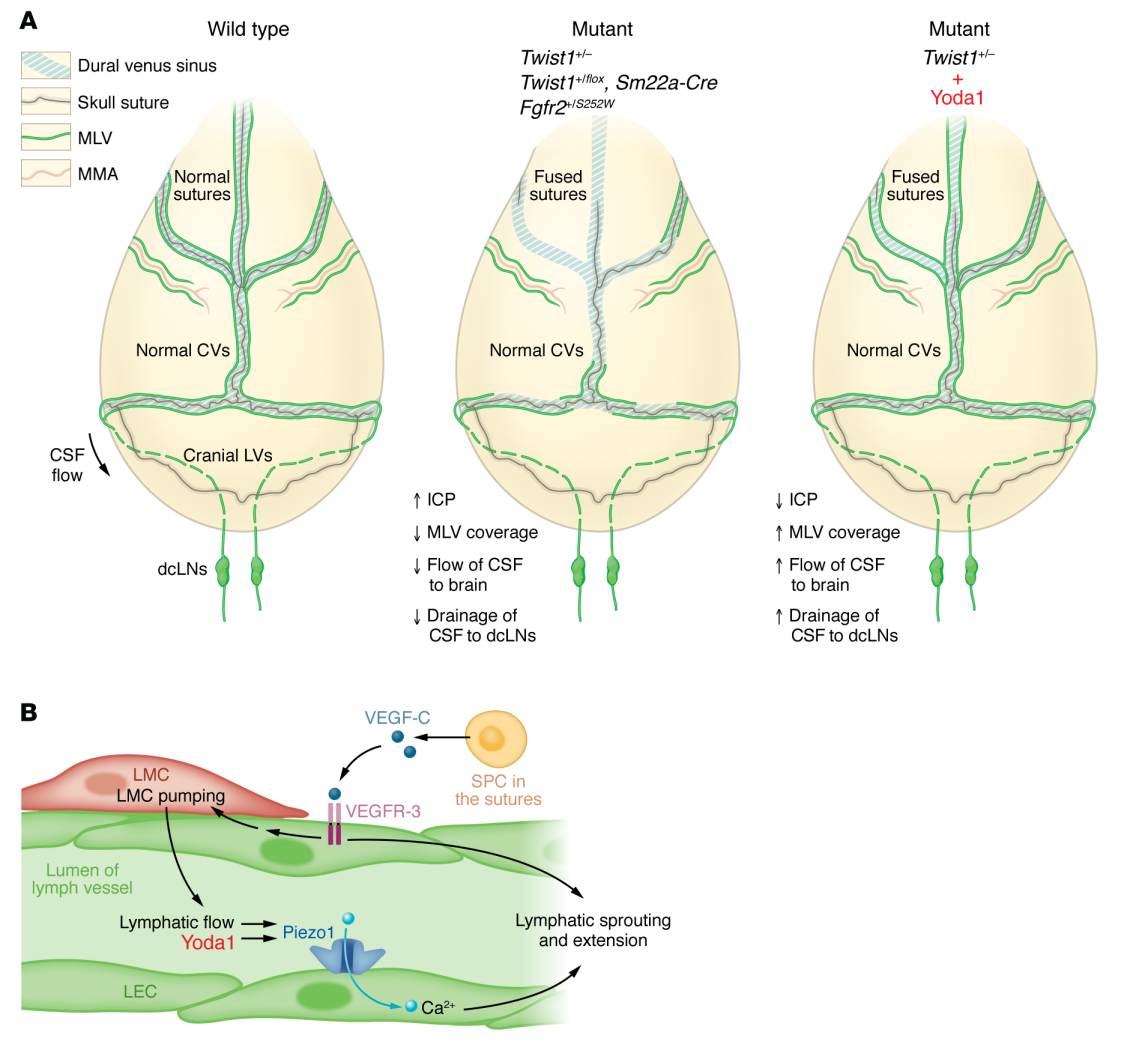
Craniosynostosis mutations and Yoda1 affect LVs, brain-CSF perfusion and outflow, and ICP. (**A**) In WT mice, CSF flows via MLVs to dcLNs along skull sutures. *Twist1^+/–^*, *Twist1^+/fl^;Sm22a-Cre,* and *Fgfr2^+/S252W^* mice display fused skull sutures, increased ICP, diminished paravascular CSF-to-brain (also known as glymphatic) flow, reduced perisinusoidal MLV coverage, and impaired CSF drainage to dcLNs, while still having normal CVs and middle meningeal artery–associated (MMA-associated) MLVs. Yoda1 treatment of *Twist1^+/–^* mice decreases ICP, restores CSF-to-brain flow, increases MLV coverage, and reinstates CSF drainage to the dcLNs (12). (**B**) Yoda1 interaction with Piezo1 facilitates the fluid flow–induced conformational changes in the Piezo1 cation channel, lowering its activation threshold mediated by calcium (Ca^2+^) influx (12). Piezo1 has also been shown to promote lymphatic endothelial cell (LEC) sprouting and expansion, functions that overlap with SPC-derived VEGF-C. VEGF-C binds to VEGFR-3, which additionally enhances lymphatic muscle cell (LMC) contractility, leading to increased lymph flow (23).

In this issue of the *JCI*, Matrongolo et al. showed that *Twist1*-haploinsufficient CS mouse models (both *Twist1^+/–^* mice and *Twist1^fl/+^;Sm22a-Cre* mice) exhibited increased ICP that correlated with dorsal MLV defects and impaired transfer of CSF tracers into MLV hotpots with poor subsequent drainage into dcLNs (12). *Twist1^+/–^* CS mice did not develop CV malformations, unlike in the more severe models, such as in the *Twist1^fl/–^;Sm22a-Cre* mice, indicating that the increase in ICP does not always require CV malformations. Similar results were also observed in a mouse model of Alpers syndrome (*Fgfr2^S252W/+^* knock-in mice that exhibit loss of ligand specificity and hyperactivity of FGFR2), indicating that pathological changes in MLVs may be conserved across multiple forms of CS (Figure 1). Interestingly, the authors also showed that only CV-associated MLVs were defective, but MLVs associated with arteries and not cranial sutures were unaffected, indicating that proximity to cranial sutures regulates artery-versus-vein–associated MLVs differentially.

Genetic ablation of MLV using verteporfin or surgical ligation of cranial LVs inhibits paravascular CSF-brain flow, also known as glymphatic flow (13). The authors confirmed that the MLV defects in CS mice were correlated with impaired CSF-brain perfusion and worsening of AD pathology when the CS mice were crossed with 5×FAD transgenic mice that accumulate β-amyloid and develop an aggressive AD-like disease.

## Yoda1 to the rescue

Piezo1 is a mechanosensitive ion channel protein that is involved in several physiological processes, including regulation of urinary osmolality, blood pressure, epithelial cell crowding, and blood vessel development (14). Recently, Piezo1 was shown to regulate lymphatic valve development (15, 16) and fluid flow–activated LV expansion, and its activation alleviated lymphedema in a mouse model (17). Genetic defects in human *PIEZO1* result in two overlapping phenotypes of generalized lymphatic dysplasia of Fotiou and dehydrated hereditary stomatocytosis with or without pseudohyperkalemia and/or perinatal edema (18).

Yoda1 is the first small molecular agonist of Piezo1 that sensitizes Piezo1 response to its external mechanical stimuli (19). Yoda1 boosts the effect of Piezo1 in LVs to unidirectional flow, which controls sprouting, expansion, and maintenance of LVs (17). Yoda1 also improves microglial phagocytosis, resulting in Aβ clearance in the 5×FAD model of AD (20). Matrongolo et al. showed that treatment of *Twist1^+/–^* mice exhibiting CS with Yoda1 reduced ICP and improved paravascular CSF-brain influx, MLV coverage, and CSF macromolecule clearance to dcLNs (Figure 1). A similar beneficial effect of Yoda1 was also observed in aged mice that had undergone atrophic and hyperplastic changes in MLVs (12). These data indicate that Piezo1 activation is beneficial in the treatment of CS-associated ICP increase and that it can also rejuvenate age-related MLV changes (12, 14).

## SPC and VEGF-C therapy alleviate CS pathology

Congruent results were recently reported by Ma and colleagues, who injected SPCs into the defective sutures of the *Twist^+/–^* mice (21). This experiment resulted in decreased ICP, improved MLV coverage, reinstated brain-CSF efflux, and normalized cognitive deficits in sociability, social memory, and motor learning, as indicated by rotarod tests. Notably, similar to muscle cells wrapping meningeal arteries, SPCs produce substantial amounts of VEGF-C, and photoablation of MLVs nullifies the therapeutic effect of SPC implantation, emphasizing the pivotal role of MLVs (4, 21). Furthermore, intrathecal injection of an AAV1 vector that produces VEGF-C lowered ICP, restored MLVs, CSF-brain influx, and interstitial fluid-CSF efflux in the *Twist1^+/–^* mice afflicted with CS, similar to Yoda1 (21).

## Conclusions

The study of Matrongolo et al. (12), along with the findings of Ma et al. (21), constitute a notable contribution to our understanding of the mechanisms responsible for increased ICP in CS, particularly in patients afflicted with the Saethre-Chotzen syndrome. Both studies indicate anatomical and functional defects in MLVs as a contributor to the CS disease pathology. Subsequent studies should elucidate the precise sequence of the pathological events in CS and the functional correlations between the CS, ICP, and MLV abnormalities. The possibility exists that MLV abnormalities and dysfunction also contribute to neurological findings in some syndromic forms of human lymphedema.

Notably, Matrongolo et al. (12) unveil that Yoda1 holds promise in enhancing MLV structure and function. Although the effects of Yoda1 are pleiotropic because the Piezo cation channels are expressed by several cell types in various tissues, it may be possible to further develop Yoda1-related compounds for more specific targeting of LVs. Such studies should also reveal whether Yoda1 can amplify the effect of VEGF-C in lymphangiogenesis, boost the effect of VEGF-C gene therapy in human lymphedema, or facilitate MLV drainage from the brain (13, 22). One could also test if VEGF-C improves the brain microglial activation by Yoda1 and whether the effect could ameliorate brain Aβ burden and cognitive impairment in 5×FAD mice (20). Overall, the groundbreaking reports showcased recently (21) and by Matrongolo et al. (12) open avenues for potential therapeutic interventions, highlighting the promising prospect of ameliorating MLV-related abnormalities and associated complications.

## Version 1 02/15/2024

Electronic publication
